# Lycopene Inhibits PRRSV Replication by Suppressing ROS Production

**DOI:** 10.3390/ijms26157560

**Published:** 2025-08-05

**Authors:** Ying-Xian Ma, Ya-Qi Han, Pei-Zhu Wang, Bei-Bei Chu, Sheng-Li Ming, Lei Zeng

**Affiliations:** 1 College of Veterinary Medicine, Henan Agricultural University, Zhengzhou 450046, China; 15238338032@163.com (Y.-X.M.); 15238368237@163.com (Y.-Q.H.); 18703808057@163.com (P.-Z.W.); chubeibei@henau.edu.cn (B.-B.C.); 2 Key Laboratory of Animal Biochemistry and Nutrition, Ministry of Agriculture and Rural Affairs, Zhengzhou 450046, China; 3 Key Laboratory of Veterinary Biotechnology of Henan Province, Henan Agricultural University, Zhengzhou 450046, China; 4 Longhu Advanced Immunization Laboratory, Zhengzhou 450046, China; 5 International Joint Research Center of National Animal Immunology, Henan Agricultural University, Zhengzhou 450046, China; 6 Ministry of Education Key Laboratory for Animal Pathogens and Biosafety, Zhengzhou 450046, China

**Keywords:** PRRSV, lycopene, ROS, inflammation

## Abstract

Porcine reproductive and respiratory syndrome virus (PRRSV), an enveloped single-stranded positive-sense RNA virus, poses a significant threat to global swine production. Despite the availability of modified live virus and inactivated vaccines, their limited efficacy and safety concerns highlight the urgent need for novel antiviral therapeutics. This study aimed to investigate the molecular mechanisms by which lycopene inhibits PRRSV replication. Initial assessments confirmed that lycopene did not adversely affect cellular viability, cell cycle progression, or apoptosis. Using fluorescence microscopy, flow cytometry, immunoblotting, quantitative real-time PCR (qRT-PCR), and viral titration assays, lycopene was shown to exhibit potent antiviral activity against PRRSV. Mechanistic studies revealed that lycopene suppresses reactive oxygen species (ROS) production, which is critical for PRRSV proliferation. Additionally, lycopene attenuated PRRSV-induced inflammatory responses, as demonstrated by immunoblotting, ELISA, and qRT-PCR assays. These findings suggest that lycopene inhibits PRRSV replication by modulating ROS levels and mitigating inflammation, offering a promising avenue for the development of antiviral therapeutics. This study provides new insights and strategies for combating PRRSV infections, emphasizing the potential of lycopene as a safe and effective antiviral agent.

## 1. Introduction

Porcine reproductive and respiratory syndrome (PRRS) is a highly contagious viral disease caused by the porcine reproductive and respiratory syndrome virus (PRRSV) [1]. Initially identified in the late 1980s, PRRS rapidly disseminated globally [2,3], inflicting substantial economic losses on the global swine industry. PRRSV is an enveloped, positive-sense, single-stranded RNA virus within the Arteriviridae family [4,5]. Pigs are the exclusive natural hosts, with porcine alveolar macrophages (PAMs) being the primary target cells in vivo. In vitro, PRRSV replicates and propagates in various cell lines, including monkey kidney cell lines such as MARC-145 and MA-104 [6,7]. Despite the development of inactivated and attenuated vaccines, these interventions have significant limitations, necessitating the pursuit of safe and effective therapeutic agents for PRRS.

Lycopene, with a chemical formula of C_40_H_56_, is a naturally occurring carotenoid predominantly found in tomatoes and other plants [8]. It is classified as a tetraterpene composed of eight isoprene units consisting solely of carbon and hydrogen atoms. Lycopene exhibits potent antioxidant [9], anti-inflammatory [10], and anticancer properties [11,12], as supported by multiple studies. Additionally, lycopene supports cardiovascular health by reducing cholesterol levels, protects against age-related macular degeneration and other ocular diseases, and promotes skin health [13]. Given its antioxidant and anti-inflammatory activities, which indirectly enhance immune function, lycopene has been proposed as a potential antiviral agent. Severe acute respiratory syndrome coronavirus 2 (SARS-CoV-2) is the primary pathogen responsible for coronavirus disease 2019 (COVID-19), mediating infection through the binding of its spike protein to the host ACE2 receptor. However, the ongoing mutations of the viruses pose serious challenges to the efficacy of the available antiviral drugs against SARS-CoV-2. Therefore, novel drugs with fantastic efficacy are always deserving of investigation. Yang et al. discovered that a nanobody named IBT-CoV144 may serve as a promising therapeutic agent. Its mechanism of action involves blocking viral recognition by the ACE2 receptor on host cells [14]. Additionally, studies suggest that naturally occurring lycopene may also have potential therapeutic effects against COVID-19 [15]. Viral infections can induce oxidative stress within host cells, triggering the innate antiviral immune response. The interaction between viral proteins and the mitochondrial membrane has been shown to increase reactive oxygen species (ROS) production. Although elevated ROS levels can activate the innate immune response, accumulating evidence suggests that excessive ROS production may facilitate viral replication [16], underscoring the therapeutic potential of antioxidant compounds. PRRSV infection induces oxidative stress by inhibiting the activity of antioxidant enzymes. The resulting oxidative stress environment appears to be conducive to viral replication.

The NLRP3 inflammasome is a critical component of the innate immune response, defending against various viral infections by regulating inflammatory pathways. Activation of the NLRP3 inflammasome and subsequent secretion of cytokines such as IL-1β and IL-18 are pivotal in initiating the host immune response, limiting viral spread, and coordinating immune cell activation. Viruses trigger NLRP3 activation through specific molecular patterns like viral RNA, DNA, or proteins. Upon recognition of these pathogen-associated molecular patterns [17], NLRP3 undergoes conformational changes and oligomerization, recruiting ASC and pro-caspase-1 to form the inflammasome. This process leads to the cleavage of pro-caspase-1 into active caspase-1, which catalyzes the maturation and secretion of pro-inflammatory cytokines IL-1β and IL-18 [18]. These cytokines are essential for recruiting immune cells, activating T cells and macrophages, enhancing inflammation, and initiating antiviral defense. The NLRP3 inflammasome is implicated in immune responses to various viruses, including influenza, herpes simplex virus, respiratory syncytial virus, and hepatitis viruses [19,20,21].

In this study, MARC-145 and PAMs were utilized as model systems to investigate the impact of lycopene on PRRSV replication. Our findings demonstrated that lycopene inhibits PRRSV proliferation by suppressing ROS production. Furthermore, lycopene mitigated the inflammation induced by PRRSV infection, underscoring its potential as an antiviral therapeutic agent.

## 2. Results

### 2.1. Effects of Lycopene on Cell Viability, Cell Cycle, and Apoptosis

**Figure 1:**
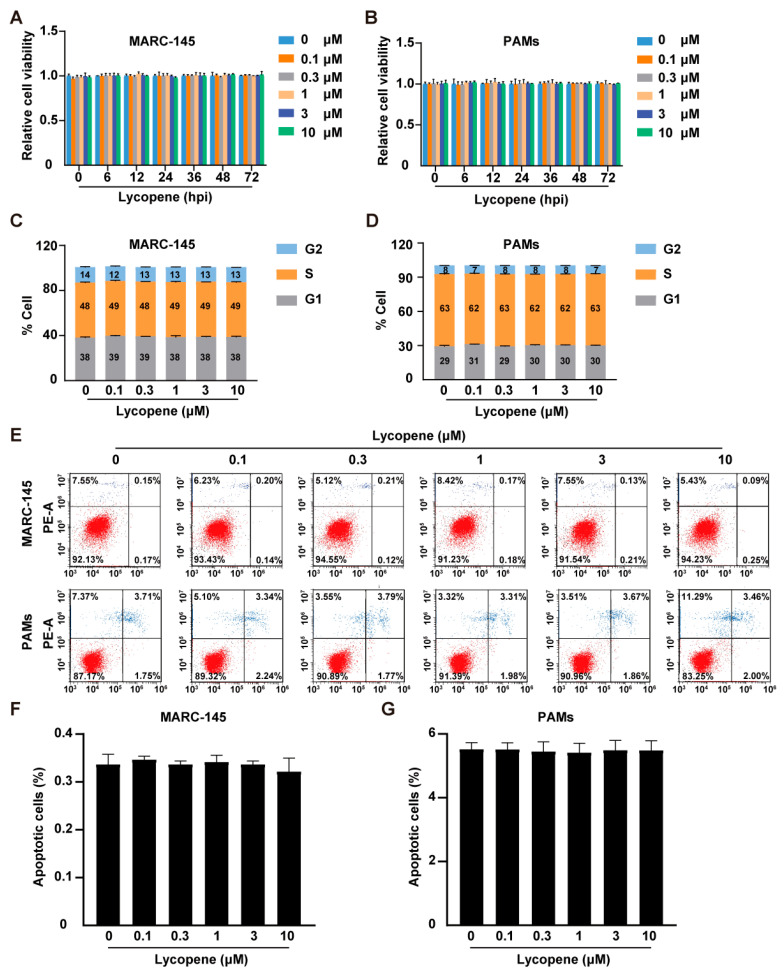
Effect of lycopene on cell viability, cell cycle, and apoptosis. (**A**,**B**) MARC-145 (**A**) and PAM (**B**) cells were treated with lycopene (0–10 μM) for 0–72 h. Cell viability was assessed with CCK-8 assay. (**C**,**D**) MARC-145 (**C**) and PAM (**D**) cells were treated with lycopene (0–10 μM) for 36 h, and the cell cycle was assessed by flow cytometry following Hoechst 33, 342 staining. (**E**–**G**) MARC-145 and PAMs were treated with lycopene (0–10 μM) for 36 h. Cell apoptosis was evaluated by flow cytometry using Annexin V-FITC and PI staining (**E**). Panels (**F**,**G**) show the percentage of cell death.

We initially evaluated the impact of lycopene on cell viability, cell cycle progression, and apoptosis. MARC-145 cells and PAMs were treated with lycopene (0–10 μM) for 0–72 h. Cell viability, assessed using the CCK-8, revealed that lycopene exerted no cytotoxic effects on either cell type (Figure 1A,B). Subsequently, cells were exposed to lycopene (0–10 μM) for 48 h, and the flow cytometry analysis of cell cycle phases indicated no significant alterations in DNA synthesis (G1, S, and G2 phases) in both MARC-145 and PAMs (Figure 1C,D). Additionally, the treatment of MARC-145 and PAMs with lycopene (0–10 μM) for 48 h and subsequent assessment of apoptosis using Annexin V/PI staining showed that lycopene did not significantly increase the percentage of apoptotic cells (Figure 1E–G).

### 2.2. Lycopene Inhibits PRRSV Infection In Vitro

**Figure 2:**
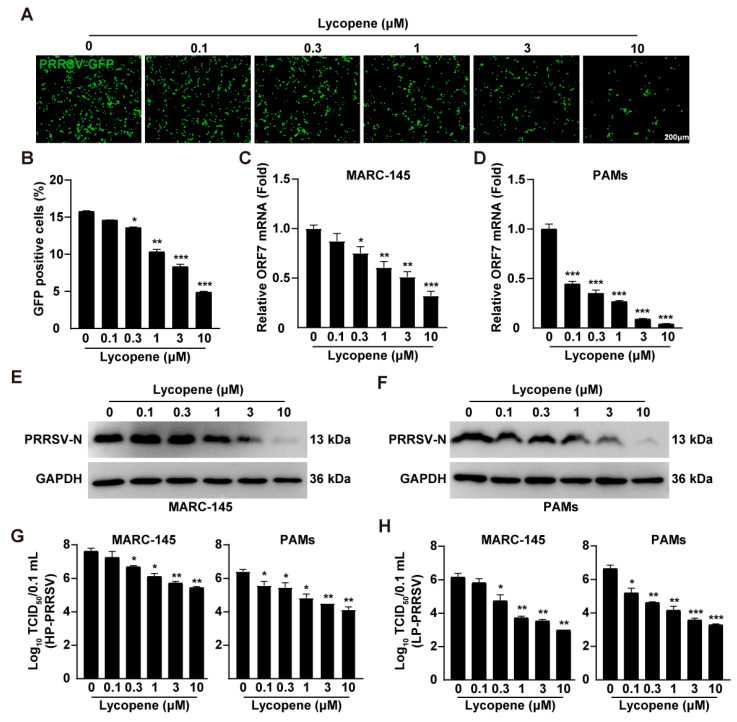
Lycopene inhibits PRRSV replication in vitro. (**A**) MARC-145 cells were infected with PRRSV-GFP (MOI = 10) and treated with lycopene (0–10 μM) for 48 h. Virus replication was observed using fluorescence microscopy. (**B**) Flow cytometry analysis of GFP-positive cells from panel (**A**). *** *p* < 0.001; ** *p* < 0.01; * *p* < 0.05 (the *p* value is calculated vs. control). (**C**,**D**) MARC-145 and PAMs were infected with HP-PRRSV (MOI = 1) and treated with lycopene (0–10 μM) for 48 h. The mRNA level of PRRSV ORF7 was analyzed by qRT-PCR. *** *p* < 0.001; ** *p* < 0.01; * *p* < 0.05 (the *p* value is calculated vs. control). (**E**,**F**) MARC-145 (**E**) and PAMs (**F**) were treated as in C, and PRRSV-N and GAPDH were detected by immunoblotting. (**G**,**H**) MARC-145 and PAMs were infected with HP-PRRSV (MOI = 10) and LP-PRRSV (MOI = 10) and treated with lycopene (0–10 μM) for 48 h. Virus titers were determined by TCID_50_ assay *** *p* < 0.001; ** *p* < 0.01; * *p* < 0.05 (the *p* value is calculated vs. control).

To determine the antiviral efficacy of lycopene against PRRSV, we employed a recombinant PRRSV-GFP strain expressing GFP to monitor viral replication [22]. Fluorescence microscopy and flow cytometry analyses demonstrated a significant reduction in GFP signals following lycopene treatment, indicating the effective inhibition of PRRSV-GFP replication (Figure 2A,B). To further validate this antiviral effect, we measured the mRNA and protein levels of PRRSV ORF7, which encodes the N protein. qRT-PCR analysis revealed that lycopene treatment significantly downregulated PRRSV ORF7 transcription in both MARC-145 and PAMs (Figure 2C,D). Additionally, lycopene treatment reduced PRRSV N protein expression in these cells (Figure 2E,F). We also evaluated lycopene’s antiviral effects against highly pathogenic PRRSV (HP-PRRSV) and low-pathogenic PRRSV (LP-PRRSV) strains. Virus titration assays showed that lycopene inhibited the progeny virus production of both HP-PRRSV and LP-PRRSV in MARC-145 and PAMs (Figure 2G,H). These results collectively indicate that lycopene possesses potent antiviral activity against PRRSV.

### 2.3. Lycopene Restricts PRRSV Entry, Replication, and Assembly

**Figure 3:**
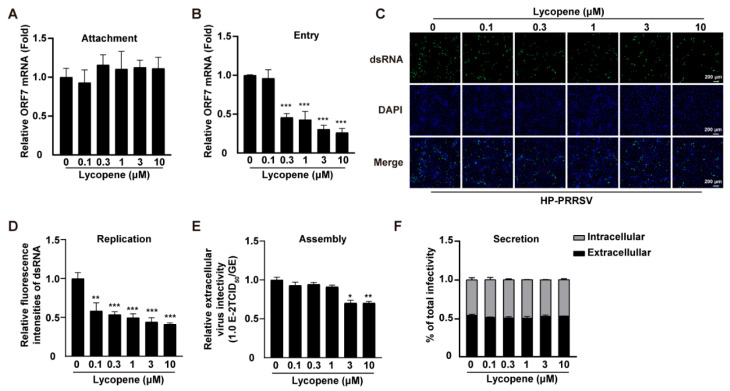
Lycopene disrupts PRRSV entry, replication, and assembly. (**A**) HP-PRRSV (MOI = 10) was incubated with MARC-145 cells at 4 °C for 1 h in the presence of lycopene (0–10 μM). After washing the cells three times with cold PBS, total RNA was extracted and reverse-transcribed into cDNA. Virus attachment was evaluated by the qRT-PCR analysis of PRRSV ORF7 mRNA. (**B**) HP-PRRSV (MOI = 10) was incubated with MARC-145 cells at 4 °C for 1 h, followed by incubation at 37 °C for 2 h in a medium containing lycopene (0–10 μM). After washing the cells three times with PBS, total RNA was extracted and reverse-transcribed into cDNA. Virus entry was evaluated by qRT-PCR analysis of PRRSV ORF7 mRNA. *** *p* < 0.001 (the *p* value is calculated vs. control). (**C**) MARC-145 cells were infected with HP-PRRSV (MOI = 10) and treated with lycopene (0–10 μM) for 36 h. Virus replication was evaluated by dsRNA staining. (**D**) Quantification of relative fluorescence intensity of dsRNA from panel (**C**). *** *p* < 0.001; ** *p* < 0.01 (the *p* value is calculated vs. control). (**E**) MARC-145 cells were infected with HP-PRRSV (MOI = 10) and treated with lycopene (0–10 μM) for 36 h. Virus assembly efficiency in the supernatant was determined by comparing the infection titer (TCID_50_/mL) with the total PRRSV genome equivalent (ORF7). ** *p* < 0.01; * *p* < 0.05 (the *p* value is calculated vs. control). (**F**) MARC-145 cells were infected with HP-PRRSV (MOI = 10) and treated with lycopene (0–10 μM) for 36 h. Virus secretion efficiency was measured by the ratio of intracellular and extracellular infectivity to total infectivity.

To elucidate the molecular mechanisms underlying lycopene’s antiviral effects, we investigated its impact on various stages of the PRRSV life cycle. Virus attachment to host cells is a crucial initial step. We co-incubated HP-PRRSV with lycopene on ice for 1 h and assessed virus–cell binding using qRT-PCR with ORF7-specific primers. The results showed equivalent ORF7 mRNA levels in MARC-145 cells, irrespective of lycopene treatment, indicating that lycopene does not affect virus attachment (Figure 3A). Next, we examined lycopene’s influence on viral entry. MARC-145 cells were incubated with HP-PRRSV on ice for 1 h, followed by a 2 h incubation at 37 °C with or without lycopene. qRT-PCR analysis revealed that lycopene impeded virus entry (Figure 3B). We also assessed PRRSV genome replication using dsRNA staining, which showed a significant reduction in dsRNA signals upon lycopene treatment, indicating inhibited viral replication (Figure 3C,D). Furthermore, the analysis of virus assembly revealed a decrease in PRRSV particle production, as evidenced by reduced infection titers and viral genome copy numbers (Figure 3E). However, the lycopene treatment did not affect the infectivity of intracellular or extracellular PRRSV, suggesting no impact on virus release (Figure 3F). These findings indicate that lycopene interferes with PRRSV entry, replication, and assembly but does not affect virus attachment or release.

### 2.4. Lycopene Inhibits PRRSV Proliferation by Suppressing ROS

**Figure 4:**
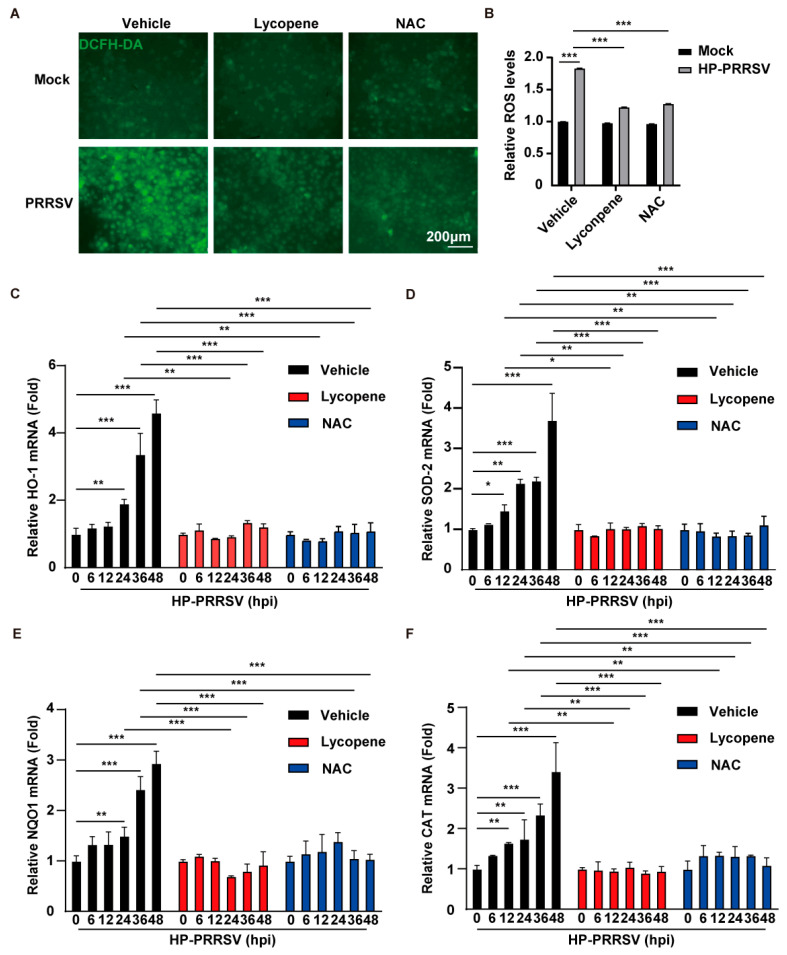
Lycopene inhibits PRRSV-induced ROS. (**A**) MARC-145 cells were infected with HP-PRRSV (MOI = 1) and treated with 10 μM of lycopene or 10 mM of NAC for 24 h. The ROS levels in the cells were determined by DCFH-DA staining. Scale bar: 200 μm. (**B**) Quantification of relative ROS levels from panel (**A**) using ImageJ (v1.8.0.345). *** *p* < 0.001 (the *p* value is calculated vs. control). (**C**–**F**) MARC-145 cells were infected with HP-PRRSV (MOI = 1) and treated with 10 μM of lycopene or 10 mM of NAC for 24 h. qRT-PCR was used to analyze the mRNA expression levels of the HO-1, SOD-2, NQO1, and CAT genes in the cells. *** *p* < 0.001; ** *p* < 0.01; * *p* < 0.05 (the *p* value is calculated vs. control).

Our previous research demonstrated that PRRSV induces oxidative stress, and lycopene is known for its potent antioxidant properties. Therefore, we investigated whether lycopene modulates oxidative stress levels in PRRSV-infected cells. DCFH-DA, a non-fluorescent dye, is converted to the fluorescent dye dichlorofluorescein (DCF) in the presence of ROS, allowing for the quantification of ROS levels using fluorescence microscopy. N-acetylcysteine (NAC), a well-established antioxidant, was used as a positive control [23]. MARC-145 cells infected with HP-PRRSV and treated with lycopene and NAC for 48 h showed enhanced DCF fluorescence signals upon PRRSV infection, indicating increased ROS levels. Lycopene treatment significantly suppressed this ROS accumulation, akin to the NAC-treated group (Figure 4A,B). Nuclear factor erythroid 2-related factor 2 (NRF2) is a transcription factor that regulates antioxidant defense genes. Elevated ROS levels trigger NRF2 translocation to the nucleus, where it binds to antioxidant response elements in target gene promoters, upregulating genes such as HO-1, SOD-2, NQO1, and CAT. Both lycopene and NAC inhibited this upregulation (Figure 4C–F). These results suggest that lycopene inhibits viral proliferation by mitigating PRRSV-induced ROS.

### 2.5. Lycopene Inhibits PRRSV-Induced Inflammatory Response

**Figure 5:**
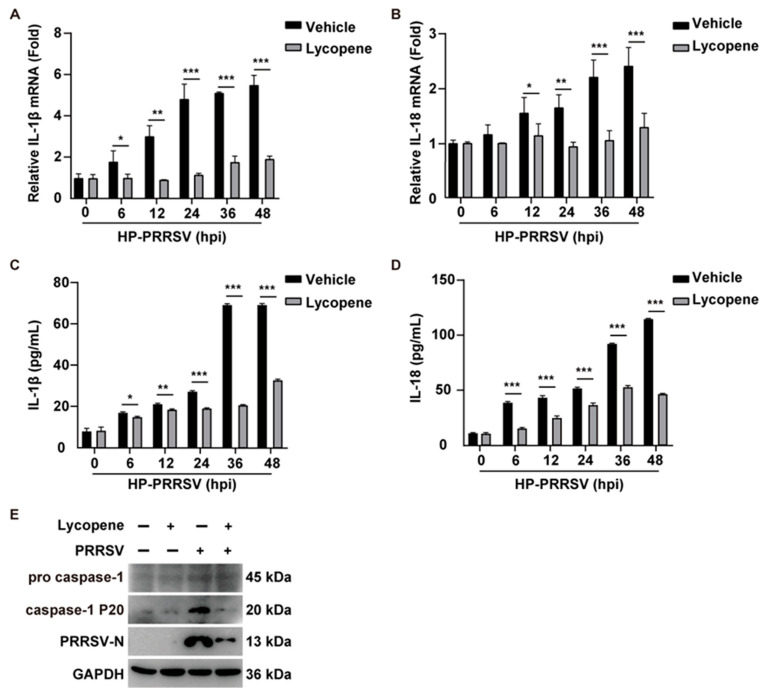
Lycopene inhibits PRRSV-induced inflammatory response. (**A**,**B**) PAM cells were infected with HP-PRRSV (MOI = 1) and treated with 10 μM of lycopene for 0–48 h. qRT-PCR analysis was conducted to evaluate the mRNA expression levels of IL-1β and IL-18 in the cells. *** *p* < 0.001; ** *p* < 0.01; * *p* < 0.05 (the *p* value is calculated vs. control). (**C**,**D**) PAM cells were infected with HP-PRRSV (MOI = 1) and treated with 10 μM of lycopene for 0–48 h. ELISA was used to measure the secretion levels of IL-1β and IL-18 in the cell supernatant. *** *p* < 0.001; ** *p* < 0.01; * *p* < 0.05 (the *p* value is calculated vs. control). (**E**) PAM cells were infected with PRRSV (MOI = 1) and treated with 10 μM of lycopene for 48 h. Immunoblotting was performed to analyze the levels of pro-caspase-1, caspase-1 P20, and GAPDH.

PRRSV infection activates the NLRP3 inflammasome, promoting the maturation and secretion of pro-inflammatory cytokines like IL-1β and IL-18, which are crucial for immune responses but can lead to excessive inflammation, or “cytokine storm”. To determine if lycopene alleviates this cytokine storm, we assessed IL-1β and IL-18 transcription using qRT-PCR. PRRSV infection significantly upregulated both cytokines’ transcription (Figure 5A,B). The ELISA confirmed the increased IL-1β and IL-18 secretion in the culture medium of PRRSV-infected PAMs (Figure 5C,D). The lycopene treatment effectively inhibited both the transcription and secretion of these cytokines (Figure 5A–D). Caspase-1 activation, essential for L-1β and IL-18 maturation, was observed in PRRSV-infected PAMs but not in lycopene-treated cells (Figure 5E). These findings indicate that lycopene mitigates the cytokine storm induced by PRRSV infection by inhibiting inflammasome activation and subsequent pro-inflammatory cytokine release.

## 3. Discussion

PRRSV is an enveloped, single-stranded, positive-sense RNA virus. Its high mutation rate leads to the emergence of multiple genotypes [24], causing significant disruptions in the global swine industry. Current prevention strategies primarily rely on modified live vaccines and inactivated vaccines [25]. However, the genetic and antigenic heterogeneity of PRRSV limits vaccine efficacy, resulting in the suboptimal performance of commercially available vaccines [26]. Consequently, there is an urgent need for alternative strategies to combat PRRSV, a priority for its prevention and control.

Recent research has led to significant advancements in PRRSV management. T. Xia and colleagues demonstrated that non-thermal plasma reactor discharge can inactivate both enveloped viruses like PRRSV and non-enveloped viruses such as bacteriophage MS2 [27]. Zhan-Ding Cui utilized high-throughput screening to identify nitazoxanide as a promising PRRSV inhibitor, targeting NMRAL1 [28]. Additionally, Cui’s team showed that caffeic acid phenethyl ester, a key component of propolis, is an effective anti-PRRSV agent [29]. Yuanqi Yang and colleagues found that ursolic acid, derived from herbs, inhibits PRRSV replication by targeting phosphatase PTPN1 and activating innate immune responses [30]. Despite these progresses, challenges such as low extraction efficiency, high synthesis losses, and high costs hinder the widespread application of these agents [31,32], necessitating the identification of more effective and cost-efficient antiviral drugs.

Lycopene, a carotenoid predominantly found in red ripe tomatoes, watermelon, grapefruit, guava, and papaya, is a highly unsaturated linear hydrocarbon. Its 11 conjugated double bonds and lack of a terminal β-ionone ring preclude pro-vitamin A activity, making it one of the most potent in vitro carotenoid antioxidants [33,34]. Lycopene exhibits antioxidant [9], anti-inflammatory [10], and anticancer properties [11]. It has also been reported to prevent cardiovascular diseases [35], improve type 2 diabetes [34], and protect against neurological disorders [36], such as Alzheimer’s disease (AD), which is a neurodegenerative disorder that manifests as memory impairment and cognitive decline [37]. Research demonstrates that LYC markedly enhances motor and cognitive functions in AD rat models [38]. As a feed additive in animal production, lycopene enhances immunity, metabolism, reproductive function, disease resistance, stress tolerance, and feed conversion efficiency while improving the quality of animal products. Given its multifaceted benefits, lycopene has been proposed as a potential antiviral agent. This study confirms lycopene’s safety for cells and demonstrates its significant ability to inhibit PRRSV proliferation.

The viral life cycle comprises five stages: adsorption, entry, genome replication, assembly, and release of progeny virus particles. To elucidate the molecular mechanisms of lycopene’s antiviral effects, this study examined its impact on the PRRSV life cycle. Epigallocatechin gallate, an antioxidant from tea leaves, inhibits PRRSV replication and assembly [39]. SP-1, an antioxidant from the marine alga Sargassum, reduces PRRSV adsorption, replication, and release by blocking the CD163 receptor [40]. This study showed that lycopene affects PRRSV entry, replication, and assembly. Lipids play a crucial role in the PRRSV life cycle, with cellular and viral lipid rafts being key factors in infection [41,42]. The inhibition of NPC1-regulated cholesterol transport blocks PRRSV replication [43], and free fatty acids are essential for viral replication [44]. PRRSV GP5 induces mitochondrial ROS to promote virus replication [45], and the virus enhances lipid synthesis via an ROS-dependent AKT/PCK1/INSIGs/SREBPs signaling axis. Antioxidant NAC treatment significantly inhibits PRRSV replication [46]. This study found that lycopene, like NAC, inhibits PRRSV-induced ROS. Excessive ROS activates the NF-κB signaling pathway and NLRP3 inflammasomes, promoting the maturation and release of IL-1β and IL-18, triggering an inflammatory cascade. Lycopene was also found to alleviate PRRSV-induced inflammatory responses.

This study demonstrates that lycopene inhibits PRRSV proliferation by suppressing ROS production, providing strong evidence for the use of antioxidants as antiviral agents. The antiviral efficacy of lycopene has been documented in multiple studies, and it is widely applied as a functional additive in veterinary medicine. Moreover, its antioxidant and anti-inflammatory properties are well established. Our findings demonstrate that lycopene not only exerts a direct antiviral effect against PRRSV but also significantly suppresses virus-induced inflammatory responses, thereby generating a dual synergistic mechanism involving both antiviral and anti-inflammatory activities. Importantly, systematic in vitro experiments confirm that lycopene maintains normal cellular viability, cell cycle progression, and apoptotic pathways at effective concentrations, thus effectively addressing the intrinsic safety limitations of traditional attenuated live vaccines. However, further investigation is required to elucidate the underlying mechanisms of lycopene’s anti-PRRSV effects in animals and to determine its optimal dosage for practical application in production.

## 4. Materials and Methods

### 4.1. Reagents and Antibodies

Lycopene (HY-N0287) was purchased from MedChemExpress (Monmouth Junction, NJ, USA), and N-acetylcysteine (NAC, IA0050) was purchased from Solarbio (Beijing, China). TRIzol Reagent (D9108B) and SYBR Premix Ex Taq (RR420A) were purchased from TaKaRa (Shiga, Japan). The anti-GAPDH (10187-2-AP) and anti-dsRNA (85780-2-RR) antibodies were purchased from Proteintech (Rosemont, IL, USA), while the anti-Caspase-1 (sc-56036) and anti-Caspase-1 p20 (sc-398715) antibodies were purchased from Santa Cruz Biotechnology (Dallas, TX, USA). The anti-PRRSV-N antibody was stored in our laboratory. Horseradish peroxidase (HRP)-conjugated goat anti-rabbit IgG (A16104) and HRP-conjugated goat anti-mouse IgG (PA1-74421) were purchased from Thermo Fisher Scientific (Waltham, MA, USA).

### 4.2. Cells and Viruses

MARC-145 (American Type Culture Collection, Manassas, VA, USA, ATCC, CRL-12231) and HEK293T cells (ATCC, CRL-11268) were cultured in Dulbecco’s Modified Eagle’s Medium (DMEM, 10566-016; GIBCO) supplemented with 10% fetal bovine serum (FBS, 10099141C; GIBCO), penicillin (100 units/mL), and streptomycin (100 µg/mL) (B540732; Sangon). PAMs were cultured in RPMI 1640 medium (61870036; GIBCO) supplemented with 10% bovine serum (GIBCO), as previously described [22]. All cells were maintained as a monolayer at 37 °C in 5% CO_2_.

The recombinant PRRSV-GFP strain was kindly provided by Professor En-Min Zhou from Northwest A&F University (Xianyang, China) [47]. The highly pathogenic PRRSV strain HN07-1 (HP-PRRSV, GenBank accession number: KX766378.1) was generously donated by Professor Gai-Ping Zhang from Henan Agricultural University (Zhengzhou, China) [48]. The less pathogenic PRRSV strain BJ-4 (LP-PRRSV, GenBank accession number: AF331831.1) was used as previously described [22].

### 4.3. Cell Viability Analysis

Cell viability was evaluated using the Cell Counting Kit-8 (CCK-8) (GK3607, DingGuo, Beijing, China). Cells were seeded in 96-well plates at a density of 1 × 10^4^ cells per well. The following day, the culture medium was replaced with DMEM supplemented with 10% fetal bovine serum, and lycopene (0–10 μM) was added. Cells were incubated for 12 to 72 h. After incubation, 10 μL of CCK-8 solution was added to each well, and the cells were further incubated at 37 °C for 3 h. Absorbance was measured at 450 nm using a microplate reader (Varioskan Flash, Thermo Fisher Scientific, Waltham, MA, USA).

### 4.4. Cell Cycle Analysis

Cells were plated in 24-well plates at a density of 1.2 × 10^5^ cells per well. The following day, the medium was replaced with DMEM supplemented with 10% fetal bovine serum (FBS) and lycopene (0–10 μM). After incubation at 37 °C for 36 h, the cells were then dissociated using trypsin-EDTA (25200072, Gibco, New York, NY, USA) and resuspended in phosphate-buffered saline (PBS) containing 5 μg/mL of Hoechst 33342. After incubation at 37 °C for 1 h, cell cycle distribution was assessed by flow cytometry using a CytoFLEX flow cytometer (Beckman Coulter, Brea, CA, USA). Data analysis was performed using FlowJo 10.8.1 software.

### 4.5. Apoptosis Analysis

Cells were seeded into 24-well plates at a density of 1.2 × 10^5^ cells per well. The following day, the medium was replaced with DMEM/10% FBS and lycopene (0–10 μM) was added, and the cells were cultured for 36 h. Apoptosis was assessed using the Dead Cell Apoptosis Kit (ZP327, ZOMANBIO, Boston, MA, USA) with Annexin V-FITC and PI. The percentage of dead cells (Annexin V and PI double positive) was measured by flow cytometry using a CytoFLEX instrument (Beckman Coulter, Brea, CA, USA). Data were analyzed using FlowJo 10.8.1 software.

### 4.6. Fluorescence Observation and Flow Cytometry Detection

Cells were plated in 24-well plates at a density of 1 × 10^5^ cells per well. Once the cell confluence reached 50%, the cells were pretreated with a medium containing lycopene (0–10 μM) for 4 h. Subsequently, the cells were infected with PRRSV-GFP at a multiplicity of infection (MOI) of 10 and incubated at 37 °C for 1 h. After infection, the medium was replaced with a maintenance medium containing the corresponding lycopene concentration, and the cells were cultured for an additional 36 h. Fluorescence was observed using a fluorescence microscope, and the percentage of cells exhibiting green fluorescence was quantified by flow cytometry using a CytoFLEX instrument (Beckman Coulter, Brea, CA, USA).

### 4.7. qRT-PCR Analysis

Total RNA was isolated from the cells using TRIzol Reagent (9108, TaKaRa, Shiga, Japan) following the manufacturer’s instructions. The RNA was subsequently reverse-transcribed into complementary DNA (cDNA) using the PrimeScript RT Reagent Kit (RRO47A, TaKaRa, Shiga, Japan). Quantitative reverse transcription PCR (qRT-PCR) was performed in triplicate using SYBR Premix Ex Taq (RR820A, TaKaRa, Shiga, Japan). Gene expression levels were normalized to ACTB (β-actin) expression. Relative quantification was calculated using the 2-△Ct method. The primers used for qRT-PCR are as follows: PRRSV ORF7-Fw: 5′-AGATCATCGCCCAACAAAAC-3′, PRRSV ORF7-Rw: 5′-GACACAATTGCCGCTCACTA-3′; porcine ACTB-Fw: 5′-CTGAACCCCAAAGCCAACCGT-3′, porcine ACTB-Rw: 5′-GACACAATTGCCGCTCACTA-3′; Monkey ACTB-Fw: 5′-CGTGGACATCCGTAAAGAC-3′, Monkey ACTB-Rw: 5′-GGAAGGTGGACAGCGAGGC-3′; Monkey HO-1-Fw: 5′-ACTCCCTGGAGATGACTCCC-3′, Monkey HO-1-Rw: 5′-AGTCTTGCGCTTTGTTGCTG-3′; Monkey SOD-2-Fw: 5′-GTGGAGAACCCAAAGGGGAG-3′, Monkey SOD-2-Rw: 5′-GCCTGTTGTTCCTTGCAGTG-3′; Monkey NQO1-Fw: 5′-CAGCGGCTCCATGTACTCTC-3′, Monkey NQO1-Rw: 5′-AGGATCTGAATTCGGGCGTC-3′; Monkey CAT-Fw: 5′-AGTGATCGGGGGATTCCAGA-3′, Monkey CAT-Rw: 5′-AAGTCTCGCCGCATCTTCAA-3′; porcine IL-18-Fw: 5′-AGGGACATCAAGCCGTGTTT-3′, porcine IL-18-Rw: 5′-CGGTCTGAGGTGCATTATCTGA-3′; porcine IL-1β-Fw: 5′-GCCCTGTACCCCAACTGGTA-3′, porcine IL-1β-Rw: 5′-CCAGGAAGACGGGCTTTTG-3′.

### 4.8. Immunoblotting Analysis

Cells were lysed in lysis buffer (50 mM of Tris-HCl, pH 8.0, 150 mM of NaCl, 1% Triton X-100, 1% sodium deoxycholate, 0.1% SDS, and 2 mM of MgCl_2_) containing a protease and phosphatase inhibitor cocktail (HY-K0010 and HY-K0022, MedChemExpress, Monmouth Junction, NJ, USA). Protein concentrations were quantified using the BCA Protein Assay Kit (BCA01, DingGuo, Beijing, China). Protein samples were resolved by SDS-PAGE and subsequently transferred to polyvinylidene fluoride (PVDF) membranes (C3117, Millipore, MA, USA). After blocking with 5% non-fat milk (A600669, Sangon Biotech, Shanghai, China) for 1 h, the membranes were incubated overnight with primary antibodies at 4 °C. The membranes were then incubated with corresponding HRP-conjugated secondary antibodies for 1 h at room temperature. Immunoblotting signals were detected using the Luminata Crescendo Western HRP Substrate (WBLURO500, Millipore, MA, USA) and visualized using a GE AI600 Imaging System.

### 4.9. 50% Tissue Culture Infectious Dose (TCID_50_) Assay

On day 0, MARC-145 cells were seeded at a density of 1 × 10^4^ cells per well in a 96-well plate. On day 1, the cells were infected with serially diluted virus (dilution range from 10^−1^ to 10^−12^) and incubated at 37 °C for 1 h. Following infection, the cells were washed with PBS to remove unbound virus. Then, 200 μL of maintenance medium (DMEM supplemented with 2% fetal bovine serum) was added to each well, and the cells were cultured for 3 to 5 days. The cytopathic effects were monitored daily, and the TCID_50_ values were determined using the Reed–Muench method.

### 4.10. Viral Attachment Assay

MARC-145 cells were seeded in 6-well plates at a density of 1 × 10^5^ cells per well. Once the cells reached approximately 60% confluence, the medium was replaced with HP-PRRSV (MOI = 1) viral solution containing varying concentrations of lycopene (0–10 μM), and the plate was incubated at 4 °C for 1 h to facilitate viral adsorption. Following adsorption, the cells were washed three times with pre-chilled PBS. Total RNA was then extracted, reverse-transcribed into complementary DNA (cDNA), and subjected to quantitative reverse transcription PCR (qRT-PCR) to quantify the PRRSV ORF7 mRNA expression levels.

### 4.11. Viral Entry Assay

The cells were inoculated with pre-chilled HP-PRRSV (MOI = 1) viral solution and incubated at 4 °C for 2 h to allow for viral adsorption. Afterward, the cells were washed three times with pre-chilled PBS, and the medium was replaced with a fresh medium containing varying concentrations of lycopene (0–10 μM). The cells were then incubated at 37 °C for 2 h. Following incubation, the cells were treated with trypsin (1 mg/mL) to remove residual virus particles from the plasma membrane. The viral genome copy number in the cells was subsequently quantified by qRT-PCR.

### 4.12. Viral Replication Assay

Cells were infected with HP-PRRSV (MOI = 10) and incubated at 37 °C for 1 h. The virus solution was then replaced with a medium containing the appropriate concentration of lycopene. After 24 h, viral replication was assessed by staining for double-stranded RNA (dsRNA).

### 4.13. Viral Assembly Assay

Cells were infected with HP-PRRSV (MOI = 10) and incubated at 37 °C for 1 h. The virus solution was then replaced with a medium containing the appropriate concentration of lycopene. After 36 h, the viral assembly efficiency in the supernatant was assessed by measuring the infection titer (TCID_50_/mL) and quantifying the total PRRSV genomic equivalents.

### 4.14. Viral Secretion Assay

Cells were infected with HP-PRRSV (MOI = 10) and incubated at 37 °C for 1 h. The virus solution was then replaced with a medium containing the appropriate concentration of lycopene. After 36 h, the samples were collected for viral titer determination. The ratio of intracellular to extracellular infectivity relative to total infectivity was calculated to assess the viral release efficiency.

### 4.15. 2′,7′-Dichlorodihydrofluorescein Diacetate (DCFH-DA) Assay

DCFH-DA is a non-fluorescent dye that can be taken up by cells in the presence of reactive oxygen species (ROS) and subsequently converted into the fluorescent compound dichlorofluorescein (DCF). The fluorescence intensity of DCF can then be measured using a fluorescence microscope to assess the ROS levels in the cells. After treatment, the cells were incubated with 10 μM of DCFH-DA (S0033S, Beyotime, Haimen, China) at 37 °C in the dark for 30 min, followed by two washes with PBS. Fluorescent images were captured using a fluorescence microscope equipped with an appropriate FITC bandpass filter. Relative fluorescence was quantified using ImageJ software (v1.8.0.345).

### 4.16. ELISA

The concentrations of IL-1β and IL-18 in the cell supernatants were quantified using ELISA kits from R&D Systems (IL-1β, PLB00B) and Advanced Biochemical (IL-18, ABCE-EL-P007).

### 4.17. Statistical Analysis

All data were analyzed in Prism 8 software (GraphPad Software, Inc, Boston, MA, USA) with Student’s two-tailed *t*-test or a one-way ANOVA. *p* < 0.05 was considered statistically significant. Data are expressed as the mean ± standard deviation from three independent experiments. Three independent replicates were performed for each experiment.

## Data Availability

The data that support the findings of this study are directly available in Mendeley Data, V1, doi:10.17632/gjh39tyc44.1 Research Data at https://data.mendeley.com/datasets/gjh39tyc44/1 (accessed on 16 July 2025).
